# Canine Placenta Recellularized Using Yolk Sac Cells with Vascular Endothelial Growth Factor

**DOI:** 10.1089/biores.2018.0014

**Published:** 2018-07-01

**Authors:** Paula Fratini, Nathia Nathaly Rigoglio, Gustavo de Sá Schiavo Matias, Ana Claudia O. Carreira, Rose Eli Grassi Rici, Maria Angelica Miglino

**Affiliations:** ^1^ Department of Surgery, Faculty of Veterinary Medicine and Animal Science, University of São Paulo, São Paulo, Brazil.; ^2^ NUCEL (Cell and Molecular Therapy Center) and NETCEM (Center for Studies in Cell and Molecular Therapy), Medical Clinics Department, School of Medicine, University of São Paulo, São Paulo, Brazil.; ^3^ Department of Biotechnology, Interunits Graduate Program in Biotechnology, Institute of Biosciences, University of São Paulo, São Paulo, Brazil.

**Keywords:** extracellular matrix, decellularization, recellularization, placenta, yolk sac cells, VEGF

## Abstract

Regenerative medicine has been growing because of the emergent need for tissues/organs for transplants and restorative surgeries. Biological scaffolds are important tools to try to solve this problem. The one used in this reserach was developed by an acellular biological scaffold from canine placenta with a rich source of cellular matrix. After decellularization, the cellular matrix demonstrated structural preservation with the presence of important functional proteins such as collagen, fibronectin, and laminin. We used cells transduced with vascular endothelial growth factor (VEGF) to recellularize this scaffold. It was succeeded by seeding the cells in nonadherent plaques in the presence of the sterelized placenta scaffold. Cells were adhered to the scaffold when analyzed by immunocytochemistry and scanning electron microscopy, both showing sprouting of yolk sac VEGF (YSVEGF) cells. This recellularized scaffold is a promissory biomaterial for repairing injured areas where neovascularization is required.

## Introduction

The necessity of tissues and organs for transplants exceeds its availability, taking to search for therapeutic alternatives.

A new tool as biological scaffolds derived from decellularized tissues and organs, more specifically extracellular matrix (ECM) may be useful in human and animal regenerative medicine as an alternative to transplantation. These scaffolds have been successfully applied in preclinical and clinical trials.^1^

The ECMs may originate from various tissues such as heart,^2,3^ blood vessels,^4,5^ skin,^6^ tendons,^7^ kidney,^8^ lung,^9,10^ and others. In order for this matrix to be considered functional, several molecules should be preserved in its structure, include collagens, glycosaminoglycans, proteoglycans, growth factors, and adhesion proteins such as laminin and fibronectin.^11^ These molecules are essential for the interaction between cell/scaffold and structural maintenance of matrix.^12^

Among various organs and tissues that can be used in regenerative medicine, there is the placenta, discarded at birth. Due to its rich parenchyma, the placenta has been used in the production of biological biomaterials.^13–15^

The different morphological placental types can be well applied to tissue engineering. In the case of the canine species, the endothelialchorial placenta is formed by maternal endothelium, interstitial lamina, trophoblast, basal lamina, and fetal endothelium.^16^ This type of placenta is found in carnivores, such as the dog,^17^ cat,^18,19^ and others.^20,21^ This placental type is characterized by the uterine epithelium. The endotheliochorial type occurs in parts of all four major subtypes of mammals, including carnivores.^16^

Placenta as biological scaffolds, independent of the species, are promising for use as decellularized as well as recellularized, due to their rich protein complexity in ECM and developed vasculature.^22^ The placental differences between species such as morphology, size, and ECM composition should be considered in possible applications in tissue bioengineering.^23,24^ Studies use the decellularized human placenta as a biomaterial for the healing wound models,^13,25^ repair cartilage degradation,^26^ liver injury,^27^ treatment of contact hypersensitivity,^28^ and repair of wound in periarticular tissue^14^ and extract of human placental matrix and growth factors as a hydrogel that effectively supports cardiomyocytes *in vitro*.^29^ Recently, the bovine placenta decellularization with possibilities of recellularization due to the vasculature preservation was characterized.^15^

Thus, the placental ECM may constitute a possible acellular bioactive biomaterial to be associated with stem cells for applications in tissue bioengineering.

Canine yolk sac cells transduced with VEGFeGFP (YSVEGF) previously characterized by our group^30^ were used to recellularize the canine placenta scaffold. These cells have endothelial progenitor characteristics and capacity to form sproutings in functional assays. This makes it an important tool for the production of scaffolds for use in regenerative medicine where tissue neovascularization is necessary.

## Materials and Methods

### Canine placenta decellularization

Placentas of female dogs with 35 days were collected from castration campaigns. The placentas were separated into maternal and fetal portions and left in 0.1% sterile sodium dodecyl sulfate (SDS) for 15 days under agitation, in a sterile environment. After, they were washed in 1% Triton X-100. Ethics Committee number 6611181016.

### DNA quantification

The genomic DNA was isolated from 30 mg of the decellularized canine placenta using the Illustra Kit (GE Healthcare). Samples were digested with Proteinase K and lysis buffer at 56°C for 2 h. They were analyzed in a spectrophotometer at 260 nm (Nanodrop; Thermo).

### Critical point and sterilization

The placentas were passed through the critical point LEICA EM CPD 300 after decellularization. At the final, the scaffolds were completely dry.

### Recellularization process

The critical-point decellularized placentas were washed in 1× phosphate buffered saline (PBS) with 0.5% antibiotic (penicillin–streptomycin) and left in ultraviolet (UV) light for 10 min. They were tested on alpha minimum essential medium (MEM) culture medium with 10% of fetal bovine serum and incubated at 37°C with 5% CO_2_ for 24 h. An amount of 5 × 10^4^ YSVEGF and YS (control) cells, previously described by our group,^30^ were plated on untreated plates (Sarstedt) inside the scaffolds for 7 days.

### Immunocytochemistry

The plates containing the YS/YSVEGF cells and scaffolds were fixed with 4% paraformaldehyde. The scaffolds were washed with PBS +0.5% Tween and incubated with the primary antibody fibronectin (Abcam) at 1:200 dilution. Then, they were washed in PBS +0.5% Tween and the secondary antibody Alexafluor 594 (Thermo Fisher). Plates were incubated with DAPI for nuclear labeling. They were analyzed on the Confocal Microscope—Olympus Fluo View 1000 (FV1000).

### Scanning electron microscopy

The decellularized and recellularized placentas were fixed in 4% paraformoldehyde. They were dehydrated in increasing concentrations of alcohol, dried in a critical point, and glued with carbon tape in metallic (sputtercoating) with golden metallizer. They were photographed in a scanning electron microscope (SEM) LEO 435VP.

## Results and Discussion

We developed an acellular biological scaffold derived from canine placenta preserving its architecture. The presence of important proteins in your constitution (collagen, laminin, and fibronectin)^31^ contributed of being recellularized with YSVEGF cells. The genomic DNA analysis showed complete scaffold decellularization using SDS and Triton^11^ (Supplementary Fig. S1). For the recellularization assay, we carry out sterilization of the scaffold, passed at critical point (Supplementary Fig. S2).

**FIG. 1.**
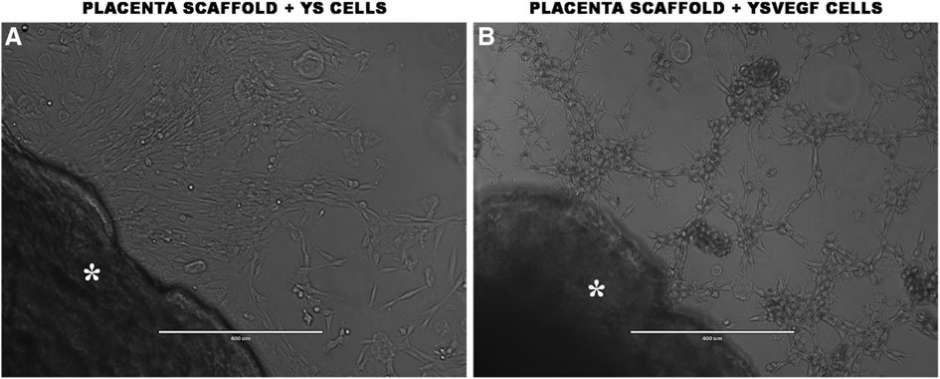
Process of placenta scaffold recellularization. In **(A),** canine YS cells on the nonadherent plate in the presence of scaffold (asterisk). Note the fibroblastoid morphology of the cells. In **(B)**, transduced YS cells with VEGF (YSVEGF) in the presence of scaffold (asterisk), there is a marked change in cellular morphology, with formation of small structured colonies and sproutings. VEGF, vascular endothelial growth factor; YS, yolk sac.

**FIG. 2.**
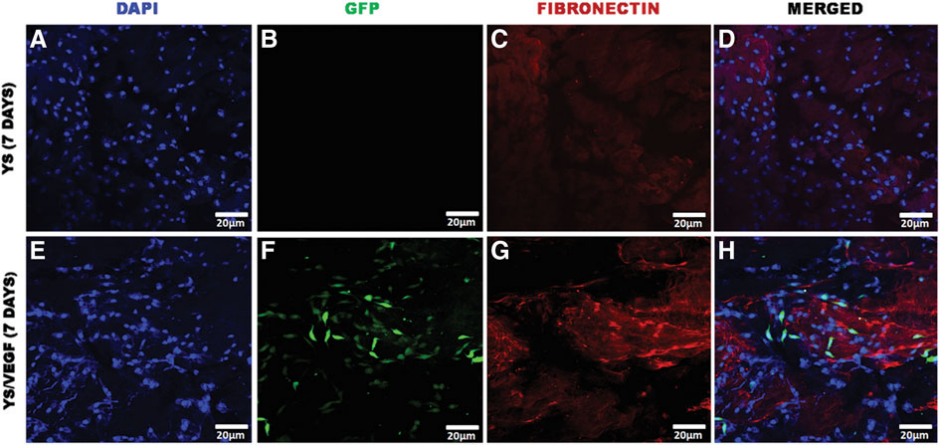
Immunocytochemistry of placental canine scaffolds with YS and YSVEGF. **(A–D)** YS cells in the presence of scaffold, in blue **(A)**, DAPI nuclei of YS cells, in **(B),** eGFP not observed, in **(C),** scaffold of placenta expressing fibronectin in red, in **(D),** YS cells in scaffold. **(E–H)** YSVEGF cells in scaffold of canine placenta, note in **(F)** the marked presence of YSVEGF cells expressing eGFP, in **(H),** the presence of these cells in the scaffold in red that expresses fibronectin is very clear, proving the efficient recellularization.

The alteration of the YSVEGF cell morphology was observed in the presence of scaffold in culture when compared to YS control cell (Fig. 1). The YSVEGF cells formed sproutings and rearranged into cord-like colonies, reminding the formation of endothelial cells. The immunocytochemical analysis confirmed the presence of adhered YSVEGF cells in the canine placenta as well as the YS cells and showed enhanced expression of fibronectin in the scaffold recellularized with YSVEGF, proving the efficacy of the method (Fig. 2). By SEM, we observed the presence of cellular colonies between scaffold fibers (Supplementary Fig. S3). Thus, proving that YS cells are a source effective for recellularization of biological scaffolds.

We observed that YSVEGF forming sproutings, a fact already demonstrated in another study of our group.^30^ This fact reinforces the idea that these cells would have the promising potential of forming a neovascularized tissue when appropriate factors and conditions are provided.

Vascular endothelial growth factor (VEGF), which is also present in YSVEGF cells, is the main regulator of angiogenesis, whose mechanism is better understood as sprouting, which is able to aid in neovascularization in the formation of new vessels.^32^

In the immunocytochemical assay, we observed overexpression of fibronectin in the scaffold that received the YSVEGF cells. According to the literature, the interaction of VEGF with ECM influences cell differentiation, modulation, and migration of cells and factors that may influence its expression.^32^ In studies where VEGF gene therapy for recovery of infarcted cardiac areas was performed, fibronectin expression increased due to cell proliferation.^33^

YS cells (YSCs) from dogs are shown to be a promising source of progenitor cells. As seen in other *in vitro* studies, they expressed Oct 3/4 (pluripotency) and cadherin protein for vascular endothelium (VE-cadherin) and when injected into immunosuppressed mice, they did not develop teratomas.^34^

YSC was also described for rodents, the immunophenotyping confirmed the mesenchymal nature of these cells (CD73^+^, CD90^+^ and CD105^+^) as well as pluripotency markers (Oct3/4 and Nanog), vascular growth (VEGF), and hematopoietic cell precursors (CD117).^20^ The functions of these structures are related to the important role that the YS have to the development of the vascular system of the embryo.^35^

There are many strategies adopted for the recellularization of biological scaffolds. Among them we can mention the use of bioreactors^36–38^ and spinners flasks^39,40^ or the immersion/agitation in detergent solutions.

We used the process of agitation and immersion in sterile SDS solutions already used for a variety of tissues. These tissues include, heart valves,^41^ skeletal muscle,^7,42^ dermis^43^ among others. On those studies, an amount of DNA below 50 ng was obtained. Also, no perfusion was necessary and the ECM structure was preserved.

Supercritical carbon dioxide removes the residues from the cells as it passes through the tissues at a rate similar to the critical point.^11^ Our samples presented a lower concentration of residual DNA and supported the sterilization of the matrix. Most importantly, the samples were not damaged. Even though, the conditions of the critical point used were not exactly the same as those of the supercritical described,^11^ we believe that the process was valid to help the efficient recellularization of the scaffold.

Our method to recellularize the scaffold, using a non adherent plate, helped the migration of the cells to the scaffold. However, some cells were still adhered to the plate. To increase cell adhesion capacity, the technique used was passive sedimentation,^44,45^ it was chosen based on the type and porosity of the scaffold.^46^ Also, the relatively long period of incubation of cells in the scaffold (7 days), and finally the presence of the VEGF and the nature of the biomaterial, may have favored the success of this first recellularization trial.

The next objective will be to test other techniques, such as rotational systems,^47–49^ which may further enhance the success of this work. The first step has already been taken.

## Conclusions

The placentas were successfully decellularized, maintaining its structural architecture. The sterilization process with UV light and critical point proved to be efficient. The YS and YSVEGF cells were adhered to the scaffold, demonstrating the ability to construct a biomaterial to be used in processes where cellular neovascularization is required.

## Supplementary Material

Supplemental data

Supplemental data

Supplemental data

## Abbreviations Used

ECM: extracellular matrix
PBS: phosphate-buffered saline
SDS: sodium dodecyl sulfate
SEM: scanning electron microscopy
UV: ultraviolet
VEGF: vascular endothelial growth factor
YS: yolk sac
YSC: yolk sac cell
