# Supplementary material for: Canine Placenta Recellularized Using Yolk Sac Cells with Vascular Endothelial Growth Factor
Source: Biores Open Access. 2018 Jul 1;7(1):101–6. doi: 10.1089/biores.2018.0014 (PMC6056259; doi:10.1089/biores.2018.0014)
Supplement: Supplemental data [file Supp_Fig1.pdf]

Supplementary Data

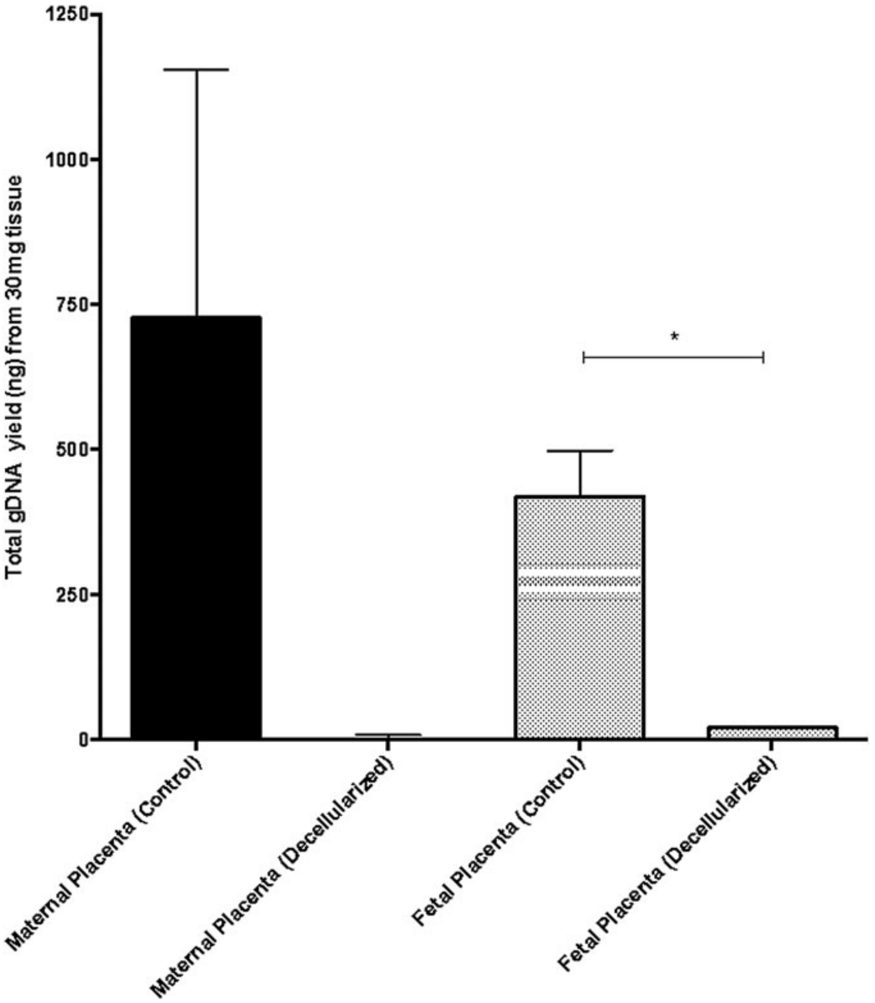

SUPPLEMENTARY FIG. S1. gDNA concentration. The graph shows that the decellularized placenta, maternal, and fetal portions had a very low expression of gDNA, being 0 ng/mg in the maternal decellularized placenta and 19.8 ng/mg in the placenta decellularized fetal portion. gDNA, genomic DNA.
