# Supplementary material for: Canine Placenta Recellularized Using Yolk Sac Cells with Vascular Endothelial Growth Factor
Source: Biores Open Access. 2018 Jul 1;7(1):101–6. doi: 10.1089/biores.2018.0014 (PMC6056259; doi:10.1089/biores.2018.0014)
Supplement: Supplemental data [file Supp_Fig2.pdf]

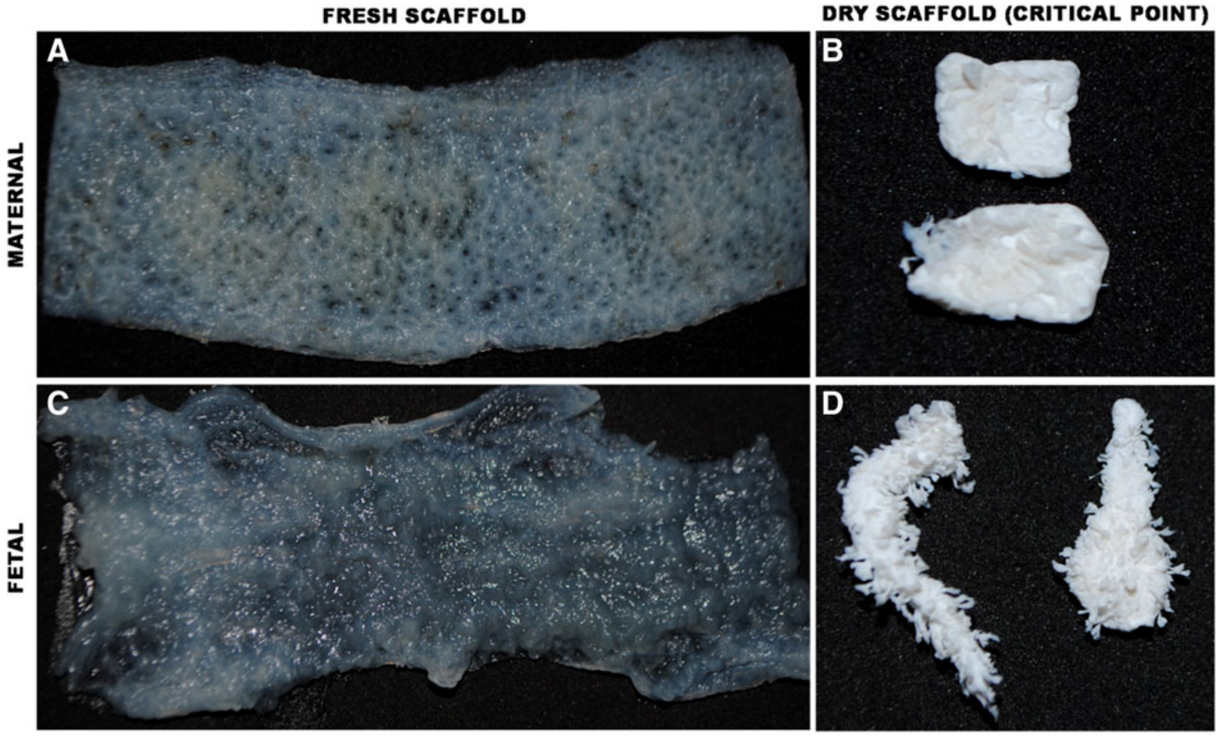

SUPPLEMENTARY FIG. S2. Decellularized canine placenta scaffolds. In **(A)**, the maternal and **(C)** fetal portions of the canine placenta are observed after the process of decellularization with 0.1% SDS for 15 days, note that the structure of the tissue are preserved and in **(B)** maternal and **(D)** fetal the same dried decellularized placentas after passing through the critical point. SDS, sodium dodecyl sulfate.
