# Supplementary material for: Canine Placenta Recellularized Using Yolk Sac Cells with Vascular Endothelial Growth Factor
Source: Biores Open Access. 2018 Jul 1;7(1):101–6. doi: 10.1089/biores.2018.0014 (PMC6056259; doi:10.1089/biores.2018.0014)
Supplement: Supplemental data [file Supp_Fig3.pdf]

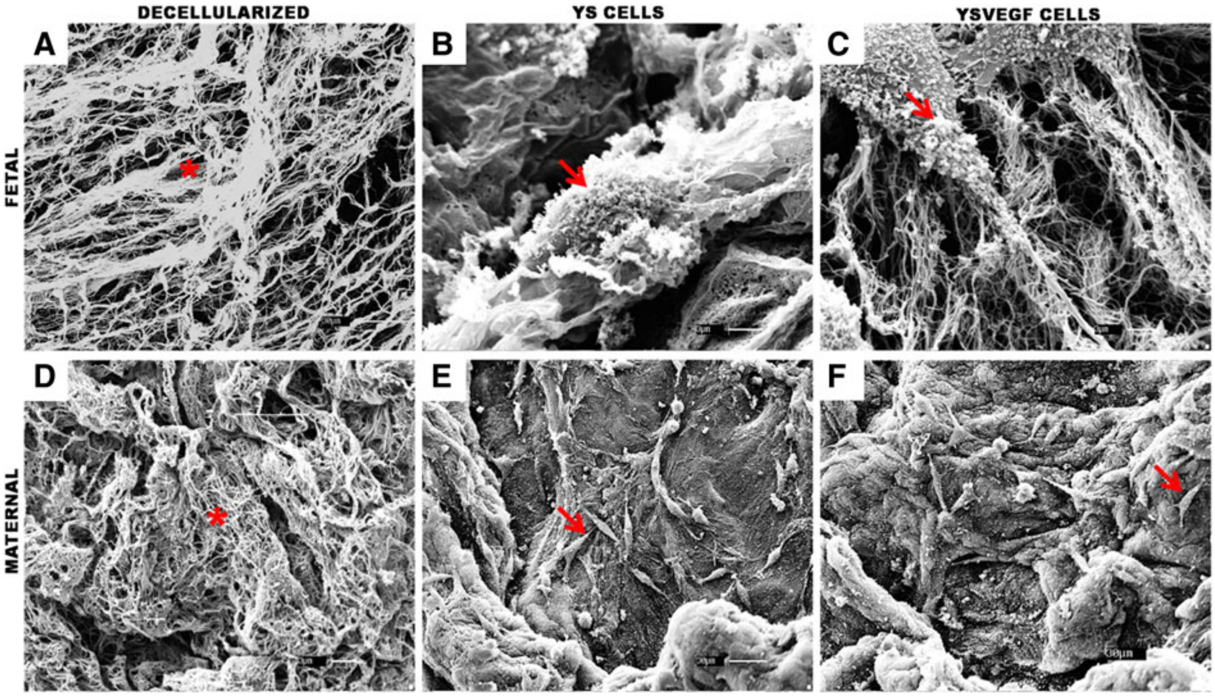

SUPPLEMENTARY FIG. S3. Scanning electron microscopy of recellularized placentas with YS and YSVEGF. In **(A, D)** control decellularized placentas without cells, only the presence of tissue fibers (asterisks) in the maternal and fetal portions is observed. In **(B, E),** the presence of fibroblastoid YS cells in the matrix (arrows) is observed, the same being observed in **(C, F)** with YSVEGF cells, arrows evidencing the numerous presence of YS cells in the placenta scaffold. VEGF, vascular endothelial growth factor; YS, yolk sac.
